# Polyphenolic Fraction from Olive Mill Wastewater: Scale-Up and in Vitro Studies for Ophthalmic Nutraceutical Applications

**DOI:** 10.3390/antiox8100462

**Published:** 2019-10-08

**Authors:** Maria Domenica Di Mauro, Giovanni Fava, Marcella Spampinato, Danilo Aleo, Barbara Melilli, Maria Grazia Saita, Giovanni Centonze, Riccardo Maggiore, Nicola D’Antona

**Affiliations:** 1 National Research Council of Italy, Institute of Biomolecular Chemistry (CNR–ICB), Via Paolo Gaifami 18, 95126 Catania, Italy; giovanni.fava@hotmail.com (G.F.); spampinatomarcella@gmail.com (M.S.); 2 MEDIVIS, Corso Italia, 171, 95127 Catania, Italy; danilo.aleo@medivis.it (D.A.); barbara.melilli@medivis.it (B.M.); mariagrazia.saita@medivis.it (M.G.S.); 3 Envisep srl–Z.Ind. VIII Strada, 29-95121 Catania, Italy; laboratorio@acimgroup.it (G.C.); riccardo.maggiore@libero.it (R.M.)

**Keywords:** olive mill wastewater, polyphenols, valorization, adsorbents, ophthalmic hydrogel, anti-inflammatory and antioxidant activity

## Abstract

The valorization of food wastes is a challenging opportunity for a green, sustainable, and competitive development of industry. Approximately 30 million m^3^ of olive mill wastewater (OMWW) are produced annually in the world as a by-product of the olive oil extraction process. In addition to being a serious environmental and economic issue because of their polluting load, OMWW can also represent a precious resource of high-added-value molecules such as polyphenols that show acclaimed antioxidant and anti-inflammatory activities and can find useful applications in the pharmaceutical industry. In particular, the possibility to develop novel nutraceutical ophthalmic formulations containing free radical scavengers would represent an important therapeutic opportunity for all inflammatory diseases of the ocular surface. In this work, different adsorbents were tested to selectively recover a fraction that is rich in polyphenols from OMWW. Afterward, cytotoxicity and antioxidant/anti-inflammatory activities of polyphenolic fraction were evaluated through in vitro tests. Our results showed that the fraction (0.01%) had no toxic effects and was able to protect cells against oxidant and inflammatory stimulus, reducing reactive oxygen species and TNF-α levels. Finally, a novel stable ophthalmic hydrogel containing a polyphenolic fraction (0.01%) was formulated and the technical and economic feasibility of the process at a pre-industrial level was investigated.

## 1. Introduction

Olive mill wastewater (OMWW) is a complex mixture of vegetation waters, soft tissues of the olive fruit, and water used during the various stages of the olive oil extraction process, characterized by its dark color, strong odor, a mildly acidic pH, and a very high inorganic and organic load [1,2]; in particular, the organic content (biochemical oxygen demand BOD 35–132 g/L, chemical oxygen demand (COD) 30–320 g/L) [3] consists essentially of sugars, tannins, polyphenols, polyalcohols, proteins, organic acids, pectins, and lipids [4].

In general, the high polyphenolic content (0.5–24 g/L) [5] makes OMWW difficult to biodegrade and a serious environmental and economic issue. Several methods are reported in the literature concerning the treatment and disposal of OMWW such as anaerobic digestion, aerobic fermentation, and composting, but all of them involve the loss or destruction of many functional compounds [6,7,8]. On the other hand, polyphenolic compounds, well-known for their beneficial effects on human health, due to their antioxidant, cardioprotective, anticancer, anti-inflammatory, and antimicrobial properties [9,10] are nowadays widely recognized as valuable molecules in pharmaceutical and nutraceutical fields [11], and in such a context, OMWW represents a really challenging bioresource. 

In particular, several studies have highlighted that hydroxytyrosol, the most abundant biophenol in OMWW acts as a free radical-scavenger and metal-chelator [12], protects against oxidative damage [13,14], inhibits the NADPH oxidase [15], the inducible form of nitric oxide synthase (iNOS), and the proinflammatory enzymes such as 5-lipoxygenase and cyclooxygenase [16], decreasing the production of nitric oxide, leukotrienes, and prostaglandins. Moreover, hydroxytyrosol is able to modulate the release of tumor necrosis factor-α (TNF-α) and other proinflammatory mediators [17,18].

Recent studies have reported that oxidative stress is involved in the pathogenesis of several eye diseases, such as ocular inflammation and dry eye disorder [19,20], therefore polyphenols could play an important role in the prevention and/or treatment of these pathologies [21].

Membrane separation, solvent extraction, resins treatment, centrifugation, chromatographic procedures, and enzymatic reactions [22,23,24,25] are different strategies reported in the literature to recover polyphenolic compounds from OMWW even if each of them presents a number of issues concerning both the industrial scalability and/or the economic and environmental sustainability.

In this paper we describe a novel route to selectively recover a polyphenolic fraction from OMWW, through a green and sustainable adsorption/desorption batch procedure, the scaling-up of the process at a pre-industrial level, and the in vitro studies to evaluate the cytotoxicity, antioxidant, and anti-inflammatory activities aimed to the formulation of a novel ophthalmic nutraceutical.

## 2. Materials and Methods

### 2.1. Materials

Fresh olive mill wastewater (OMWW) from Cerasuola cultivar were freshly collected in Menfi (Agrigento, Italy) in the middle-late of the olive oil processing season from a continuous three-phase olive oil processing mill operating at a malaxing temperature of 27 °C; samples were stored in airtight screw-capped tanks at −20 °C. 

Folin–Ciocalteu reagent, trifluoroacetic acid (TFA), 3-[4,5-dimethyl(thiazol-2-yl)-3,5-diphenyltetrazolium bromide (MTT), 2’,7’-dichlorofluorescein diacetate (DCFH-DA), lipopolysaccharide (LPS) and all standards were purchased from Sigma-Aldrich (Milan, Italy). All solvents were purchased from Carlo Erba (Milan, Italy). The adsorbents Purosorb™PAD428, Purosorb™PAD550, and Purosorb™PAD900 were obtained from Purolite. Statens Seruminstitut Rabbit Cornea (SIRC) cells were purchased from the American Type Culture Collection (Rockville, MD, USA). Fetal Bovine Serum (FBS), Eagle’s Minimum Essential Medium (EMEM), Phosphate Buffered Saline solution (PBS), and Penicillin-Streptomycin (5000 U/mL) were obtained from Gibco-BRL Life Technologies. *Escherichia coli* Formamidopyrimidine-DNA Glycosylase (Fpg) FLARE™ Module (4040-100-FM) was purchased from Trevigen. Rabbit Anti-TNF alpha antibody, Rabbit Anti-beta Actin antibody and Goat Anti-Rabbit IgG H&L (HRP) were purchased from Abcam. Super Signal West Pico Chemiluminescence detection system was purchased from Thermo Scientific (Rockford, IL, USA). The SkinEthic™ HCE (human corneal epithelium) tissues were purchased from Episkin (Lyon, France).

### 2.2. OMWW Pretreatment

Cerasuola-OMWW samples were centrifuged at 4000 rpm (2688 g) for 20 min to remove any solid residues of drupes and leaves and the supernatant was filtered through filter paper under vacuum condition as reported by Fava et al. (2017) [26]. Filtered OMWW were subjected to a flash-freezing process to avoid degradation of polyphenolic compounds and to ensure long-term stability and reproducibility of analyses. Samples stored at −20 °C into airtight screw-capped containers showed good stability for over 1 year; all analytical operations were performed, when possible, under argon or nitrogen as suggested by Obied et al. (2005) [27], and samples were treated preventing any alterations or contaminations by the environment.

### 2.3. Adsorption/Desorption Treatment

An aliquot of the chosen adsorbing material (Purosorb™PAD428, Purosorb™PAD900, and Purosorb™PAD550–10 g) was introduced in a column (3 × 50 cm), washed with a mixture of acetone/water (50/50) and then rinsed with water; bed column volumes amounted to 14 mL, 11 mL, and 15 mL respectively for Purosorb™PAD428, Purosorb™PAD900, and Purosorb™PAD550. The column was charged with filtered OMWW (10 mL) and eluted with pure water (50 ml) to collect the unabsorbed fraction. Subsequently, 50 mL of the chosen eluent was used to elute the column. Preliminarily different organic eluents or water/organic eluent mixtures were tested for polyphenols desorption, including methanol, ethanol, tetrahydrofuran, and ethyl acetate; in all cases, the best results were obtained with a water/ethanol (50/50) solution with a flow of 0.5 mL/min. The evaluation of maximum adsorption capacity for each resin was achieved by increasing the OMWW load volume. In order to be regenerated after use, the adsorbents were washed with ethanol (50 mL), dried, and kept at ambient temperature. Adsorbents were tested by consecutive adsorption/desorption cycles to define their recycling features.

### 2.4. Determination of Total Phenol Content

Total phenols were determined according to Di Mauro et al. (2017) [28]. Microplate spectrophotometer reader (Synergy HT multi-mode microplate reader, BioTek, Milano, Italy) was used to determinate the absorbance at λ 750 nm, and values compared against a gallic acid calibration curve (*y* = 0.002*x* + 0.030, *R*^2^ = 0.9997). Results were expressed as g/L of gallic acid.

### 2.5. Determination of Total Carbohydrates

Total carbohydrates were determined by the Dubois method [29]. The absorbance values were evaluated at 490 nm and compared against a glucose calibration curve (*R*^2^ = 0.999) from 1 to 100 mg/L (Cary UV Agilent Technology). Results were expressed as mg/L of glucose.

### 2.6. Determination of the Pollutant Load

Evaluation of the pollutant load of OMWW and eluted fractions was performed according to EPA (U.S. Environmental Protection Agency) methods by the following parameters: Chemical oxygen demand (COD, EPA test Method 410.3), biochemical oxygen demand (BOD5, EPA test Method 5210), total phosphorous (EPA test Method 365.3) and total nitrogen (EPA test Method 352.1).

### 2.7. Chromatographic Analysis of Polyphenols

HPLC-DAD (HITACHI) using a Kinetex C-18 (4.6 × 250 mm, 5 µm) column (Phenomenex) with a security guard cartridge (Phenomenex) was used for the analytic separation of polyphenols. The column temperature was maintained at 30 °C. Water (A) and acetonitrile (B) were used as eluents, both added with 0.1% trifluoroacetic acid (TFA). Samples were eluted according to the following gradient: 100% A as starting condition for 5 min; 58% A in 25 min; 100% B in 15 min, maintained for 5 min; flow rate 0.8 mL/min. Chromatograms were acquired at 280 nm. Identification of polyphenols was performed by comparison of UV spectra and retention times with the corresponding analytical standards: hydroxytyrosol, tyrosol, 1,2-dihydroxybenzene, oleuropein, 3-(4-hydroxyphenyl)propionic acid, syringic acid, 4-hydroxyphenylacetic acid (PHPA), 4-hydroxybenzoic acid, *p*-coumaric acid, *tran*s-ferulic acid, gallic acid monohydrate, vanillic acid, caffeic acid, and verbascoside. For each commercial standard a 5-points calibration curve was used for the quantification.

### 2.8. H-NMR Analysis

Bruker Avance TM 400 spectrometer at 400.13 MHz was used to record ^1^H-NMR spectra. All previously dried samples were solubilized in deuterium oxide (D_2_O). Chemical shifts (δ) are given as parts per million relative to the residual solvent peak.

### 2.9. Adsorption Equilibrium Tests

Six different aliquots of the adsorbent material were weighted and placed in six beakers, to which 25 ml of OMWW were added. The quantities of adsorbent material were chosen in such a manner that the six beakers contained an adsorbent mass to OMWW volume ratio equal to 1:12, 1:10, 1:8, 1:6, 1:4, and 1:2, respectively. Thus the weighted masses of adsorbent were, in all cases, 2.08 g, 2.5 g, 3.12 g, 4.17 g, 6.25 g, and 12.5 g. The mixtures were stirred for 2 h and then let rest overnight, at 20 °C and in darkness, in order to reach the adsorption equilibrium. Samples were then filtered on 0.45 µm filters and analyzed for their content in Total Polyphenols and COD.

### 2.10. Adsorption Kinetic Tests

A weighted aliquot of adsorbent was placed in a beaker together with a proper volume of OMWW and subjected to continuous stirring. The adsorbent mass to the OMWW volume ratio, chosen on the base of previously obtained equilibrium adsorption data, was 10 g of adsorbent for 50 mL of OMWW. Small aliquots of the mixture (1 mL each) were sampled at predetermined time intervals, and analyzed for their total polyphenols content.

### 2.11. Cycling Efficiency Tests

A weighted aliquot of adsorbent was placed in a beaker together with a proper volume of OMWW and subjected to continuous stirring. The adsorbent mass to OMWW volume ratio, as in the kinetic tests, was 1:5. After a time of 30 min the mixture was filtered on a porous septum, the filtrate was collected, and the adsorbent was washed with water and placed again in the beaker, this time together with a hydroalcoholic solution (50% ethanol). After 15 min of stirring, this mixture was filtered, collected the filtrate phase, and then washed the adsorbent with water, to submit it to a new cycle. This sequence of operations was repeated 5 times. Total polyphenols concentration was determined in all filtrates, calculating the adsorption and desorption efficiency for each cycle.

### 2.12. Process Scale-Up

A prototype pilot plant was developed to scale up the laboratory process. Purosorb™PAD428 was used as an adsorbent phase since it gave the best results in the laboratory phase as described in the “Results and Discussion” section. Since residual oils and sand present in the input waters can cause problems of clogging, accumulation, and eroding or obstructing pipes and machinery, the first stage was the de-oiling and sand removal; a steel tank in which OMWW was released with a flow rate of 0.5 m^3^/h and working simultaneously as a settler and oil-separator was used. De-oiled OMWW was then conveyed into a storage tank, and forced with a flow rate of 300 L/h, through a sand filter, in order to retain particles with a diameter >50 µm not intercepted in the previous sedimentation step. 

Afterward, OMWW were subjected to an adsorption process on Purosorb™PAD428, with the aim to adsorb polyphenols; the system consisted of a steel vessel with a volume of 100 L, partially filled with 80 L (about 32 kg) Purosorb™PAD428. The filtered effluent flowed at a flow rate of 200 L/h, until 160 L of water was measured by a flowmeter, in accordance with the OMWW/ Purosorb™PAD428 ratio optimized in laboratory tests (5.0 ml OMWW/g stationary phase).

The obtained de-phenolized OMWW were addressed to a storage tank, which was used for different cosmeceutical applications [30]. After the adsorption phase, pure water was used to wash in countercurrent Purosorb™PAD428. Adsorbed polyphenols were than desorbed with 50 L water/ethanol/isopropanol 50:42:8 *v*/*v* (ethanol/isopropanol 85/15 used to prepare the eluent phase represented the purest commercially available composition for semi-industrial use) with a flow rate of 5 L/min, and stored in a 2000 L tank. As the last step, Purosorb™PAD428 was washed with 50 L ethanol/isopropanol 85/15, and then with water to eliminate alcoholic residues before restarting the cycle. Analytic control and chemical characterization on outputs from the various steps of the process was achieved by sampling points at various parts of the plant for each cycle sequence.

### 2.13. In Vitro Study

#### 2.13.1. Cell Cultures and Treatments

SIRC cells (passage: 18) were cultured in a 12- or 96-multiwell microplate and/or in 25 cm^2^ flasks, according to the type of assay, with EMEM supplemented with 1% penicillin-streptomycin and 10% fetal bovine serum, and incubated under a humidified atmosphere at 37 °C with 5% CO_2_. When cells reached 70% of confluence, the lyophilized fraction PAD428-FR2 was dissolved in culture medium at the appropriate final concentrations for each biological assay.

#### 2.13.2. MTT Assay

The potential cytotoxic effect of fraction was evaluated by MTT assay [30,31] on SIRC cells (2.5 × 10^4^ cells/well) untreated and treated with different concentrations of PAD428-FR2 (0.01%, 0.02%, 0.05%, 0.1%) for 24 h; subsequently, 200 μL of MTT (0.5 mg mL^−1^) in culture medium were added to each well and incubated for 3 h at 37 °C keeping a humidified atmosphere with 5% CO_2_. Finally, the supernatant was aspirated off and 100 μL of DMSO was added to each well to dissolve the formazan crystals.

A microplate spectrophotometer reader (Synergy HT multi-mode microplate reader, BioTek, Milano, Italy) at λ = 550 nm was used to measure the optical density (OD). Results were expressed as a percentage of cell viability with respect to untreated control viable cells, whose value was equal to 100%.

#### 2.13.3. Lactic Dehydrogenase Release

Lactic dehydrogenase (LDH) release was evaluated on SIRC cells untreated and treated with different concentrations of PAD428-FR2 (0.01%, 0.02%, 0.05%, 0.1%) for 24 h, measuring spectrophotometrically in the culture medium and in the cellular lysates, at λ = 340 nm by analyzing NADH reduction [32]. The percentage of lactic dehydrogenase release was calculated as the percentage of the total amount, considered as the sum of the enzymatic activity present in the cellular lysate and that in the culture medium.

#### 2.13.4. Alkaline Comet Assay

The alkaline comet assay was performed on SIRC cells untreated and treated with PAD428-FR2 (0.01%, 0.02%, 0.05%) for 24 h. The minigels were prepared as described by Tomasello et al. (2017) [33]. Then, as described by Di Mauro et al. (2017) [34], the alkaline version of the comet assay was performed. An analysis of fifty nucleoids for each sample was carried on by using an epifluorescence microscope (Leica, Wetzlar, Germany) equipped with a camera. DNA damage was evaluated by using CASP (1.2.2) image analysis software. Results were expressed as the percentage of fragmented DNA present in the comet tail (%TDNA).

#### 2.13.5. Reactive Oxygen Species (ROS) Determination

ROS levels were evaluated using 2’,7’-dichlorofluorescein diacetate [35]. A microplate spectrofluorometer reader (Synergy HT multi-mode microplate reader, BioTek, Milano, Italy) was used to measure the fluorescence (λexcitation = 488 nm and λemission = 525 nm). The total protein content was evaluated for each sample according to Bradford (1976) [36]. The results were expressed as fluorescence intensity per mg protein.

#### 2.13.6. Protective Effect against Oxidative Stress

SIRC cells were pretreated with PAD428-FR2 0.01% for 24 h and then stimulated with H_2_O_2_ (200 µM) for 10 and 30 min. Untreated cells were used as negative control; cells treated with H_2_O_2_ (200 µM) were used as a positive control. After treatments, cell viability was evaluated by MTT assay as previously described. 

The oxidative DNA damage was evaluated by the Fpg-modified comet assay [37]. After lysis, each sample was incubated with 100 µL of enzyme dilution buffer or Fpg enzyme solution in a humidity chamber at 37 °C for 45 min. Then the samples were electrophoresed in alkaline solution (300 mM NaOH, 1 mM Na_2_EDTA, pH > 13) for 20 min at 0.7 V/cm. After staining with SYBR green, the nucleoids were analyzed as already described.

ROS levels were evaluated using 2’,7’-dichlorofluorescein diacetate [35] as previously reported.

#### 2.13.7. Protective Effect against Inflammation

SIRC cells were pretreated with PAD428-FR2 0.01% for 2 h and then stimulated with lipopolysaccharide (LPS 1 μg/mL) for 24 h. Untreated cells were used as negative control; cells treated with LPS (1 μg/mL) were used as a positive control. After treatments, cell viability was evaluated by MTT assay as previously described. 

TNF-α protein expression was determined by Western Blot analysis. After treatments, proteins were extracted from SIRC cells as described by Anfuso et al. (2017) [38]. Protein samples (30 μg/lane) were subjected to SDS-PAGE and, after transferring to nitrocellulose membranes, were incubated with antibody against TNF-α and β-actin overnight at 4 °C followed by incubation with horseradish peroxidase conjugated secondary antibody, goat anti-rabbit for TNF-α and β-actin. Super Signal West Pico Chemiluminescence detection system was used to visualize the protein expression after washing with TBS-T. β-actin was used as the loading control. Image J software (Version1.43, Broken Symmetry Software, Bethesda, MD, USA) was used to analyze bands.

### 2.14. Studies for Ophthalmic Nutraceutical Application

#### 2.14.1. Ophthalmic Formulation and Stability Study

PAD428-FR2 0.01% was formulated in a hydrogel whose composition is shown in Table 1. 

Initially, three different solutions (A, B, and C) were prepared as described above. Solution A was prepared in an appropriate container (1 L) by dissolving 11.5 g of glycerol in 700 g of purified water. Next, 2 g of Carbopol^®^980 and 0.1 g of Pemulen™RT1-NF were added to the previous solution left under stirring for 2 h to ensure the complete dissolution of polymers. The resulting solution was sterilized in autoclave at 121 °C for 20 min. Solution B was prepared by dissolving 2.5 g of Na_2_HPO_4_ 12 H_2_O and 0.7 g of NaOH in 200g of purified water. Solution C was obtained by dissolving 0.2 g of EDTA and 0.10 g of lyophilized PAD428-FR2 in 79.7 g of purified water. Both solutions B and C were filtered through a hydrophilic filter PES Durapore 0.22 μm in a sterile environment and transferred into glass containers preliminarily sterilized. Solution B was added dropwise to the polymeric solution (A) stirring for 30 min. Finally, solution C was added to the obtained gel stirring for 60 min. The formulation was divided into 2 mL sterile polypropylene vials with screw cap and "O" silicone ring (Axygen^®^). All operations were carried out at 25 °C and in particular the process of filtration and closure was performed under sterile condition using a vertical laminar flow cabinet with HEPA filters (Bio Activa Plus, Aquaria^®^).

The pH, osmolality, appearance, and hydroxytyrosol concentration (%) were evaluated on different aliquots of hydrogel stored in dark conditions into 2 mL sterile polypropylene vials at 25 ± 2 °C with 60% ± 5% relative humidity (R.H.) for 3 months.

#### 2.14.2. Ocular Irritation Test

The ocular irritation potential of the formulation was evaluated by using SkinEthicTM HCE model according to the protocol and instructions of the manufacturer. Positive (ethanol-treated) and negative (PBS-treated) controls were used. The tissues were evaluated for cell viability (CV) using the MTT assay [39]. If the percentage of CV was >60%, the substance can be predicted as non-irritant (UN GHS classification: no category); if the percentage of CV was ≤60%, the substance can be considered irritant (UN GSH classification: Category 1 or Category 2).

### 2.15. Statistical Analysis

All results were obtained by three independent experiments performed in triplicate; the means and standard deviations for each value were calculated. One way ANOVA was used to assess statistical differences among different treatments. Bonferroni test was performed to obtain post hoc comparison. *p* < 0.05 as minimum level of significance was applied. Graph Prism version 5 and/or Microsoft Excel was used to perform all the analyses.

## 3. Results and Discussion

### 3.1. Selective Recovery of Polyphenolic Fraction from OMWW

Cerasuola-OMWW characterization was performed as described in previous works [26,28] and results are summarized in Table 2.

A valuable use of olive mill wastewaters could be obtained through the valorization of the recovered polyphenolic fraction. Practically, this is a hard task since biophenols are not very stable and can easily get oxidized, hydrolyzed, polymerized, conjugated, and/or complexated in this aqueous environment containing all the reactants (such as metals, enzymes, oxygen, and polysaccharides), required for these kinds of transformations. In a preliminary attempt to identify new, eco-sustainable, and cheaper materials, that are able to selectively adsorb the phenolic fraction (or a fraction of it), three “untraditional” materials were tested: corncob (the maize central core), coffee husk (a waste of the coffee torrefaction process), and volcanic ashes. These materials were chosen both because of their abundant presence in Sicily, and since they constitute wastes (in the case of volcanic ashes, wastes generated by nature itself), and were characterized by relevant disposal costs and/or polluting issues. All tested processes with these materials, regardless of the used conditions, did not give any significant results showing no selective or unselective adsorption property toward the compounds contained in OMWW. In a further attempt, three different commercial polymeric adsorbents (Purosorb™PAD428, Purosorb™PAD900, and Purosorb™PAD550) were tested separately to establish the most suitable to retain completely or partially the phenolic fraction in a selective way. 

Different eluents and values of flow or temperature were tested to find a good compromise that would allow the ability to minimize possible oxidation reactions and achieve equilibria between the various phases. The general best conditions were a flow of 0.5 mL/min at 22 °C, and elution with water, to collect the unabsorbed fraction (FR1), followed by a solution of water/ethanol (50/50) in the attempt to selectively desorb the polyphenolic fraction (FR2), and finally with pure ethanol to wash and recycle the adsorbent phase. Analysis of different eluted fractions by HPLC-DAD indicated that only Purosorb™PAD428 and Purosorb™PAD900 were able to completely retain the phenolic compounds. In fact, both aqueous fractions (PAD428-FR1 and PAD900-FR1) presented a slight yellow color and were almost completely deprived of any phenolic component as demonstrated both by chromatograms at 280 nm and by ^1^H-NMR spectra (data are shown in Supplementary materials Figures S2–S8); the results reported in Table 2 show that the eluted aqueous fractions (PAD428-FR1 and PAD900-FR1) had a very low amount of polyphenols (0.40 and 0.38 g/L). Furthermore, the values of COD and BOD were reduced by 40% and 30% respectively, and a slight decrease in the inorganic load was observed too (Table S1 shown in Supplementary material). On the other hand, high amounts of carbohydrates (not retained by the adsorbent resins) suggest that this fraction could find valuable applications in cosmetic field as described in a recent work [30].

**Figure 1:**
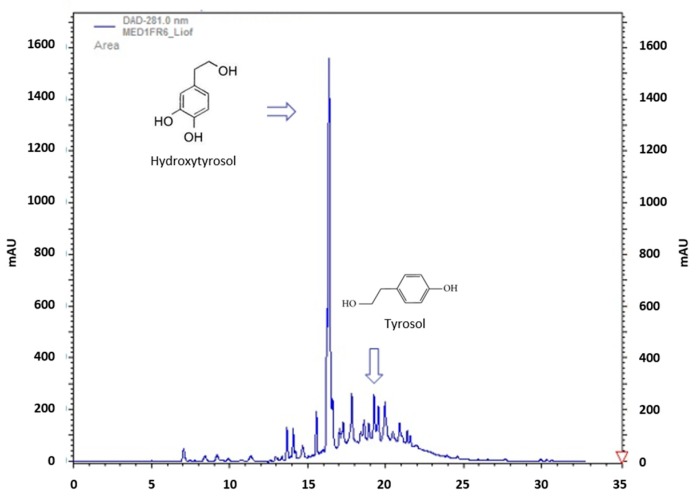
Chromatogram of PAD428-FR2 fraction at 280 nm.

Conversely, the fractions eluted with the ethanolic mixture (PAD428-FR2 and PAD900-FR2) presented an intense red–brown color and gave again similar results in terms of polyphenols content (up to 4.12 g/L, recovery 80%) as shown by the PAD428-FR2 sample chromatogram reported in Figure 1, with hydroxytyrosol and tyrosol respectively 0.90–0.85 g/L and 0.10–0.08 g/L as reported in Table 2. However other biophenols were identified in PAD428-FR2 samples as shown in Table S2 (see Supplementary materials).

The maximum volume of Cerasuola-OMWW that can be absorbed by 10 g of both adsorbent resins, before saturation, was 30 mL. Since Purosorb™PAD428 and Purosorb™PAD900 showed very similar adsorbent features, the subsequent adsorption equilibrium tests and scale-up process were carried out employing only the resin Purosorb™PAD428, and biological assays and formulation procedure of the ophthalmic nutraceutical were realized with the corresponding polyphenolic fraction (PAD428-FR2).

### 3.2. Adsorption Equilibrium and Kinetic Tests

To describe the equilibrium state in the adsorption system, the concept of dynamic equilibrium is commonly used. The liquid/gas molecules striking on the surface of a solid material can be adsorbed or rebounded; the rate of adsorption at the beginning is elevated since the adsorption sites are all available, but it decreases over time as the surface gets covered by adsorbate molecules. Conversely, the desorption rate increases because a greater number of molecules rebound until reaching the equilibrium between the adsorption rate and the desorption rate [40]. There are several equilibrium isotherm models but the most used and important in the field of adsorption for environmental cleanup are Freundlich and Langmuir isotherms [41,42].

**Figure 2:**
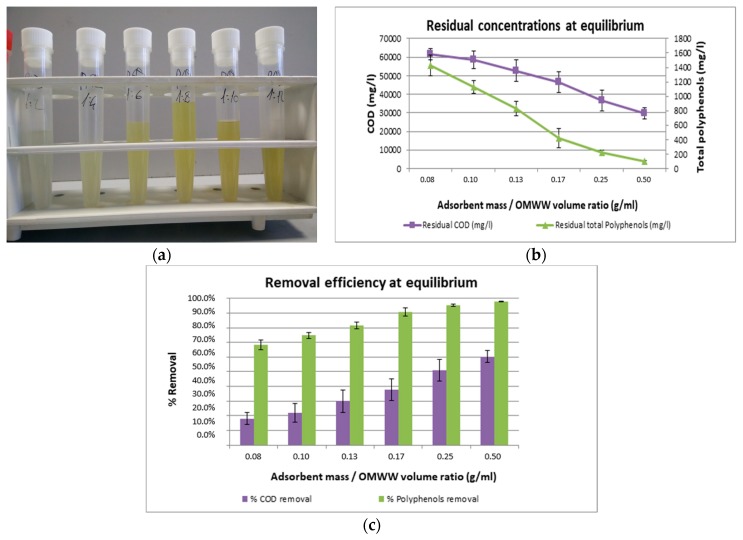
(**a**) Filtered solutions of OMWW, resulting from batch tests with Purosorb™PAD428 resin. (**b**) Residual concentrations of chemical oxygen demand (COD) and total polyphenols, at adsorption equilibrium, for the Purosorb™PAD428 resin. (**c**) Removal efficiency for COD and total polyphenols, at adsorption equilibrium, for the Purosorb™PAD428 resin. Mean values (*n* = 5) ± SD were calculated.

After adsorption equilibrium was reached, batch test mixtures were filtered (on filter paper) and collected in test tubes. The resulting solutions for Purosorb™PAD428 resin test are shown in Figure 2a. The color diminution, that corresponds to the increase in the adsorbent dose (from right to left), was a clear symptom of the removal of the polyphenolic components (that are accounted for the typical reddish OMWW color). The solutions were analyzed, with respect to their content in COD and total polyphenols, and the results (see Figure 2b) confirmed the visual interpretation.

According to Figure 2c, a significant increase in total polyphenols removal efficiency was observed when the adsorbent dose to OMWW ratio increased from 1:12 to 1:2. The total polyphenols removal reached 90% at 1:6 ratio (0.17), while the removal rate increase was less pronounced at higher ratios. This result is easily understandable since an increase in the amount of adsorbent material will increase the total surface area and the available adsorption sites, lowering the driving force for intra-particle adsorption at each adsorption site, and resulting in a minor utilization of the adsorption capacity. This leads to the conclusion 1:5 ratio is considered to be a good compromise between removal efficiency and employed quantity of adsorbent.

**Figure 3:**
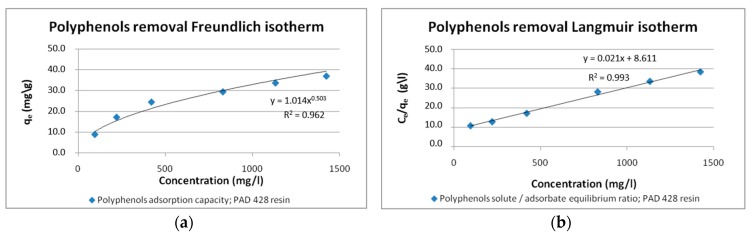
Freundlich (**a**) and Langmuir (**b**) isotherm curve for polyphenols adsorption, at equilibrium, 373 for the Purosorb™PAD428 resin. The y-axis reports: for the Freundlich curve, the equilibrium adsorption capacity "q_e_” (q_e_ = x/m, in which x = mass of adsorbate and m = mass of adsorbent); for the Langmuir curve, the "C_e_/q_e_" ratio (in which C_e_ is the solute concentration at the equilibrium).

The isotherm of total polyphenols adsorption onto Purosorb™PAD428 resin is depicted in Figure 3a, in which the *R*^2^, associated with the Freundlich model fitting, is indicated. It is observed that the curve is concave downward, indicating a favorable isotherm leading to high adsorption capacity, as depicted in the values of Langmuir constants. In the fixed-bed adsorption column, a strongly favorable isotherm would also lead to a short mass transfer zone [43].

As reported in Figure 3b, a better representation of results is obtained by the Langmuir isotherm model. This was an expected result since, compared to Freundlich isotherm, this model is more flexible in modeling adsorption from highly concentrated water solutions. This result validates the assumption of monolayer homogenous adsorption of total polyphenols on polymeric resin.

The correlation coefficient R^2^ shown in Table 3 gives privilege for Langmuir isotherm over Freundlich one. The maximum Langmuir capacity Q^0^ matches the equilibrium adsorption capacity curve (Figure 3b).

Langmuir separation factor R_L_ resulted to be equal to 0.084, indicating that the adsorption isotherm of total phenol onto Purosorb™PAD428 resin is favorable (0 < R_L_ < 1). R_L_ values give evidence that the process is reversible, supporting the hypothesis that physical adsorption occurs in this process. 

### 3.3. Cycling Efficiency Tests

One of the topics of adsorption systems is the number of cycles an adsorbent can undergo, which could be a limiting factor for the suitability and the overall economic convenience of the process. 

**Figure 4:**
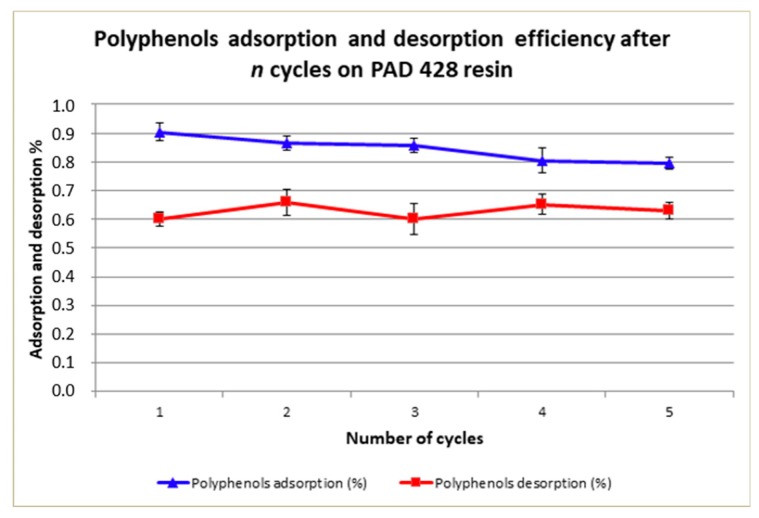
Polyphenols adsorption and desorption efficiency on Purosorb™PAD428 resin related to working cycle number. Mean values (*n* = 5) ± SD were calculated.

Cycling tests were performed on Purosorb™PAD428 resin and Figure 4 shows results: it is confirmed, for the first cycle, that adsorption efficiency, after a 30 min contact time at 1:5 adsorbent mass to OMWW volume ratio, was about 91%, as found out in previous tests. After each cycle, this value slightly decreased down to about 79% after five cycles. Concerning the desorption efficiency values, these remained quite constant throughout the several cycles with values between 60–66%. Surprisingly, the incomplete polyphenols desorption from resin adsorption sites seems not to drastically influence its adsorption efficiency, maybe because it was charged at its maximum capacity during the tests. Anyway, this is an aspect that should be further investigated in future studies.

**Figure 5:**
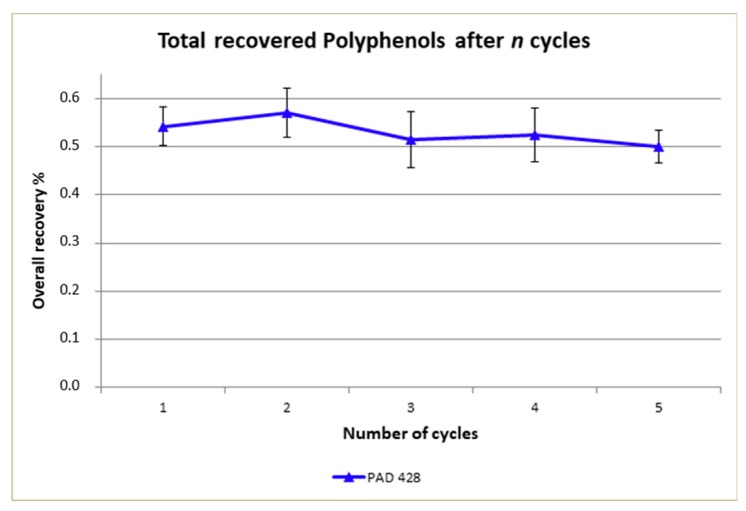
Total polyphenols recovered from OMWW using Purosorb™PAD428, related to the numbers of adsorption/desorption cycles. Mean values (*n* = 5) ± SD were calculated.

Figure 5 reports the overall polyphenols recovery. The recovery efficiency reduction, after five cycles, was of −7.5% (from 54% to 50%) for Purosorb™PAD428, even if a statistical analysis will be necessary for a better evaluation of data.

### 3.4. In Vitro Study and Ophthalmic Nutraceutical Application

Even if natural compounds and extracts are commonly considered free from harmful effects possible safety problems cannot be excluded [30]. Generally, irritation and cytotoxicity tests are performed to reduce the ocular risk of exposure to dangerous substances. Historically, animal tests such as the in vivo Draize rabbit assay were used to define the level of ocular toxicity by application of a test compound to a live rabbit’s eye and then evaluation of the biological response. Recently, several in vitro alternative techniques have been developed as the result of ethical reconsideration of the animal used for toxicology studies [44].

For this purpose, we performed an in vitro study by using SIRC cells, a cell line having a mixed epithelial and fibroblastic nature [45], already used for toxicology studies in ophthalmic field [46,47].

**Figure 6:**
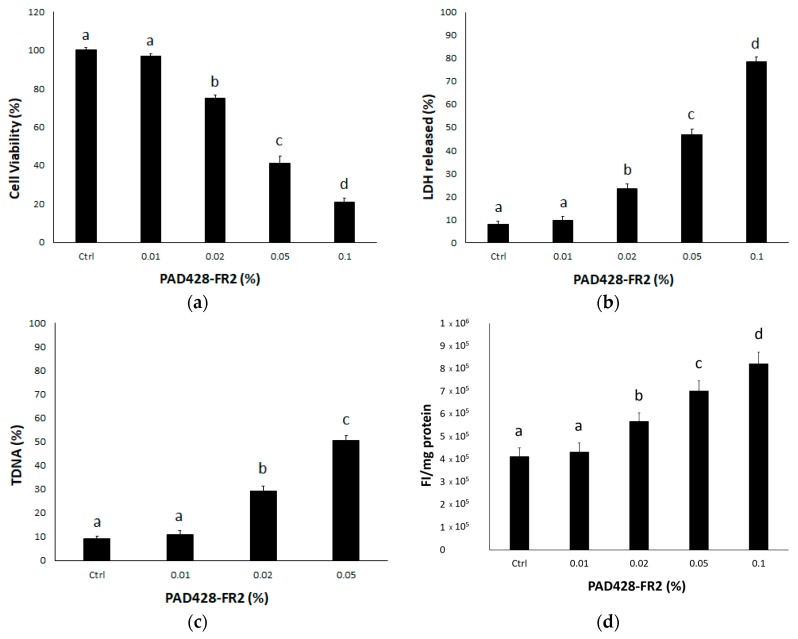
Effects of PAD428-FR2 on SIRC cells treated for 24 hours. (**a**) The results of 3-[4,5-dimethyl(thiazol-2-yl)-3,5-diphenyltetrazolium bromide (MTT) assay are expressed as the percentage of cell viability with respect to untreated control cells (Ctrl). (**b**) Lactic dehydrogenase (LDH) released. (**c**) DNA damage evaluated by alkaline comet assay; the results are expressed as the percentage of DNA present in the comet tail (%TDNA). (**d**) Reactive oxygen species (ROS) levels evaluated spectrofluorometrically by using 2’,7’-dichlorofluorescein diacetate (DCFH-DA); the results are expressed as fluorescence intensity (FI) per mg protein. Values are mean ± SD of three experiments in triplicate. Bars with different letters are significantly different (*p* < 0.05).

Our preliminary interest was to evaluate in vitro the possible cytotoxic effect elicited by the fraction PAD428-FR2. Figure 6a shows the results of MTT assay, a colorimetric method measuring the reduction of MTT, a yellow-colored tetrazolium salt, to a purple formazan by the mitochondrial dehydrogenase enzyme of living cells [30,32]. The MTT assay data obtained on SIRC cells treated with different concentrations of PAD428-FR2 for 24 hours provided evidence to show that the lowest concentration (0.01%) did not influence the cell viability compared to untreated control cells. Conversely, the treatment with higher concentrations (0.02%, 0.05%, and 0.1%) caused a drastic decrease in cell viability in a dose-dependent manner with respect to both untreated control cells and 0.01% treated cells; in particular, these concentrations (0.02%, 0.05%, and 0.1%) were able to induce a dose-dependent necrotic effect as a result of cell membrane disruption (Figure 6b), in accordance with MTT assay data.

In addition, we evaluated in vitro the potential genotoxic effects of the fraction. Among the different approaches used to study DNA damage, comet assay is a simple and fast method to assess different types of DNA damage at the single-cell level [48]. In particular, we performed alkaline comet assay that permits to identify DNA double-strand breaks, single-strand breaks, alkali-labile sites, DNA-DNA/DNA-protein cross-linking, and incomplete excision repair sites [49]. The results, shown in Figure 6c, provided evidence that the treatment with 0.02% and 0.05% induced DNA damage in a dose-dependent manner.

In order to evaluate the possible mechanism of action, we measured ROS levels by using DCFH-DA, a non-fluorescent molecule that can spread through the cell membrane and, once inside the cell, is enzymatically hydrolyzed by an intracellular esterase to non-fluorescent DCFH; then ROS are able to oxidize DCFH to the fluorescent dichlorofluorescein (DCF), whose fluorescence intensity (FI) is proportional to the level of intracellular ROS [50].

In our opinion, the observed toxic effects could be related to the ROS overproduction (Figure 6d), that can determine the accumulation of oxidized intracellular macromolecules influencing the cell viability up until inducing cell death [51].

Based on these results we decided to continue the study by excluding the highest concentrations (ranging from 0.02% to 0.1%) because of their toxicity for the selected cell line.

It is well known that oxidative stress is involved in the pathogenesis of several eye diseases, such as ocular surface inflammation and dry eye disease [19,20]. So we evaluated, for the first time to our knowledge, the antioxidant and anti-inflammatory activities of polyphenolic fraction obtained from OMWW on SIRC cells.

**Figure 7:**
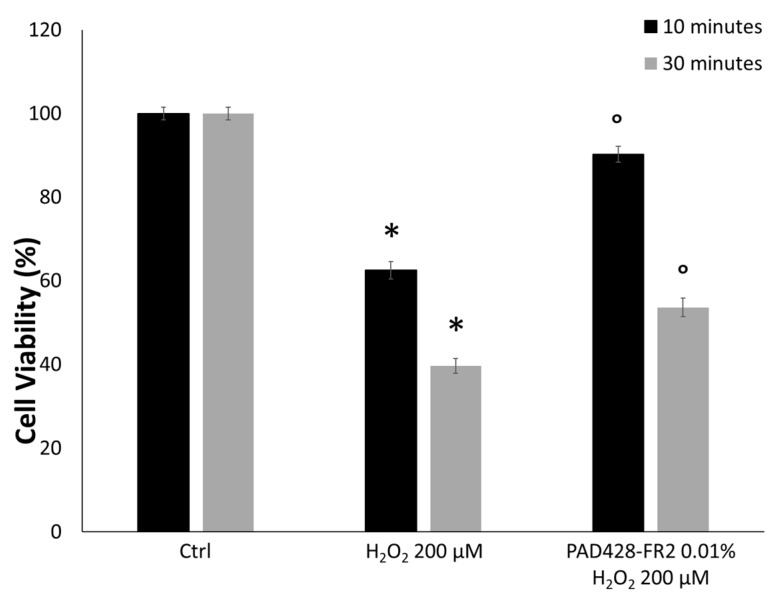
MTT assay performed on SIRC cells pretreated with PAD428-FR2 0.01% for 24 h and then stimulated with H_2_O_2_ (200 µM) for 10 and 30 min. Untreated cells were used as negative control; cells treated with H_2_O_2_ (200 µM) were used as a positive control. The results are expressed as the percentage of cell viability with respect to untreated control cells (Ctrl). Values are mean ± SD of three experiments in triplicate. * *p* < 0.05 vs. control group; ° *p* < 0.05 vs. H_2_O_2_-treated cells.

**Figure 8:**
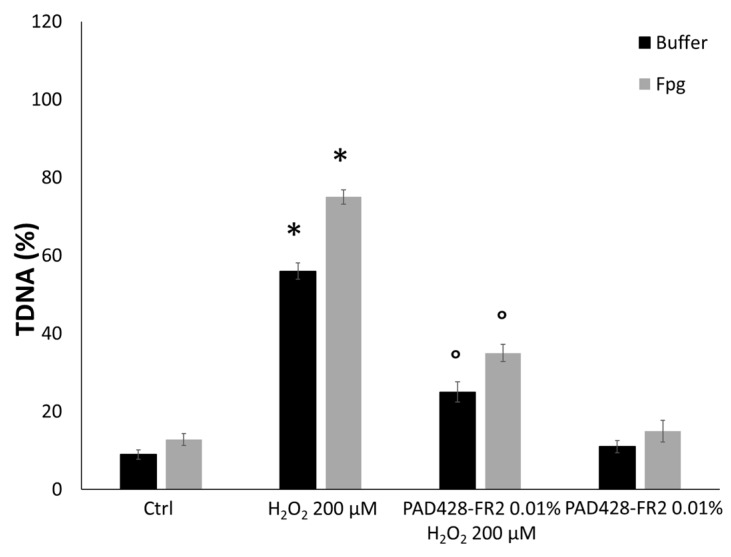
DNA oxidative damage evaluated by Fpg-modified comet assay performed on SIRC cells pretreated with PAD428-FR2 0.01% for 24 h and then stimulated with H_2_O_2_ (200 µM) for 30 min. Untreated cells were used as a negative control; cells treated with H_2_O_2_ (200 µM) were used as a positive control. The results are expressed as the percentage of DNA present in the comet tail (%TDNA). Values are mean ± SD of three experiments in triplicate. * *p* < 0.05 vs. control group; ° *p* < 0.05 vs. H_2_O_2_-treated cells.

To test the antioxidant activity, SIRC cells were pretreated with PAD428-FR2 0.01% for 24 h and then were stimulated with H_2_O_2_ (200 µM) for 10 and 30 minutes. The results of the MTT assay (Figure 7) showed that H_2_O_2_-treatment reduced cell viability compared to untreated control cell in a time-dependent manner. Moreover, it can be observed that the pretreatment with PAD428-FR2 0.01% determined an increase in cell viability with respect to H_2_O_2_-treated cells, suggesting a good protective activity of the fraction against the oxidant stimulus. In addition, we performed the alkaline version of the comet assay by using Fpg enzyme that recognizes and cuts the sites corresponding to oxidized guanine bases [37]. The results, shown in Figure 8, evidenced that the pretreatment with PAD428-FR2 0.01% was able to protect DNA from oxidative stress induced by H_2_O_2_ added for 30 min. 

**Figure 9:**
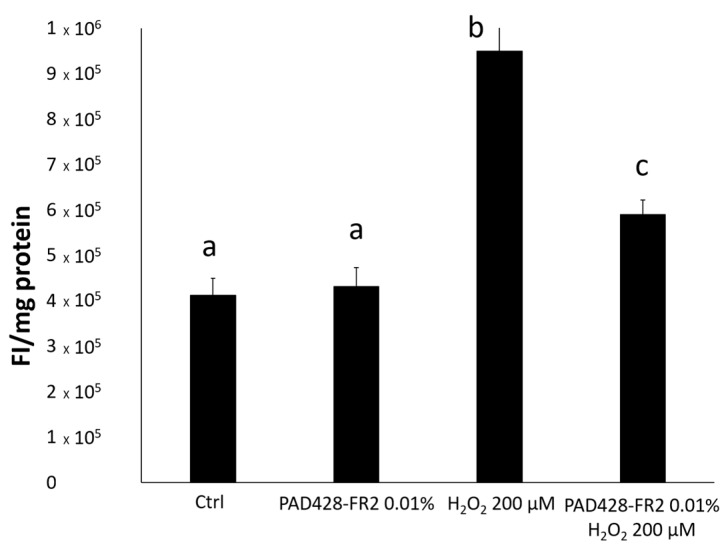
ROS levels evaluated on SIRC cells pretreated with PAD428-FR2 0.01% for 24 hours and then stimulated with H_2_O_2_ (200 µM) for 30 minutes. Untreated cells were used as a negative control; cells treated with H_2_O_2_ (200 µM) were used as a positive control. The results were expressed as fluorescence intensity per mg protein. Values are mean ± SD of three experiments in triplicate. Bars with different letters are significantly different (*p* < 0.05).

As expected, a significant rise of ROS levels was observed in H_2_O_2_-treated cells with respect to untreated control cells in a time-dependent manner. This increase was counteracted by pre-incubating cells with PAD428-FR2 0.01% (Figure 9).

Our results are in accordance with Schlupp et al. (2019) who reported the antioxidant activity of a phenol-enriched OMWW extract was able to reduce the formation of free radicals in vitro [52]. Similar results were obtained by Schaffer et al. (2007) who demonstrated that hydroxytyrosol-rich olive mill wastewater extract was able to protect brain cells from oxidative damage [53]. 

Regarding the anti-inflammatory activity, SIRC cells were pretreated with PAD428-FR2 0.01% for 2 h and then were stimulated for 24 h with LPS (1 μg/ml), the major constituent of the outer membrane of Gram-negative bacteria able to elicit inflammation. 

**Figure 10:**
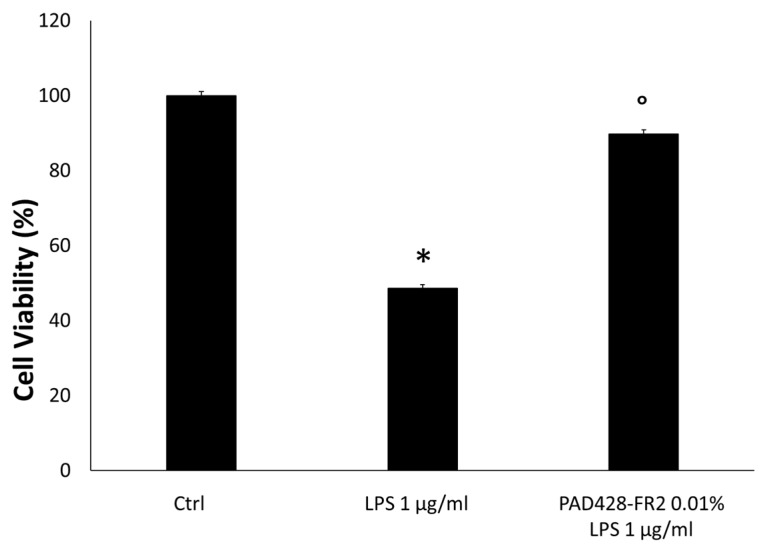
MTT assay performed on SIRC cells pretreated with PAD428-FR2 0.01% for 2 h and then stimulated with lipopolysaccharide (LPS) (1 μg/mL) for 24 h. Untreated cells were used as a negative control; cells treated with LPS (1 μg/mL) were used as a positive control. The results are expressed as the percentage of cell viability with respect to untreated control cells (Ctrl). Values are mean ± SD of three experiments in triplicate. * *p* < 0.05 vs. control group; ° *p* < 0.05 vs. LPS-treated cells.

**Figure 11:**
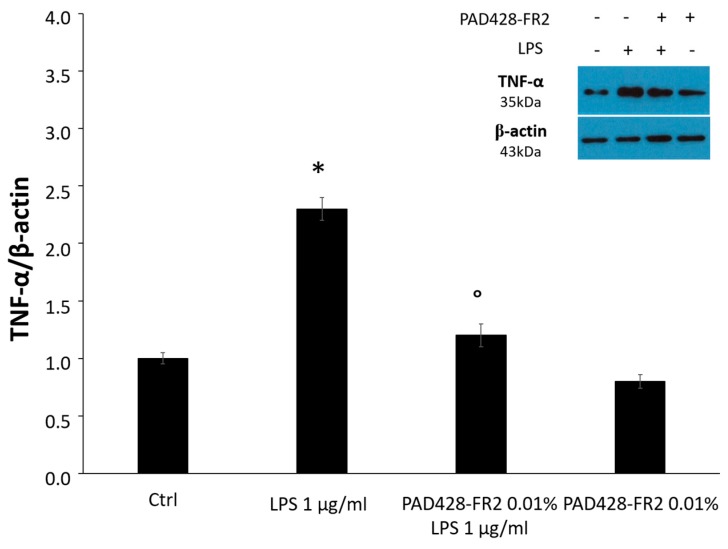
TNF-α levels evaluated by Western Blot analysis performed on SIRC cells pretreated with PAD428-FR2 0.01% for 2 h and then stimulated with LPS (1 μg/mL) for 24 h. Untreated cells were used as a negative control; cells treated with LPS (1 μg/mL) were used as a positive control. β-actin was used as the loading control. Values are mean ± SD of three experiments in triplicate. * *p* < 0.05 vs. control group; ° *p* < 0.05 vs. LPS-treated cells.

As expected, MTT data and immunoblots, shown in Figure 10 and Figure 11, revealed that LPS-treatment significantly reduced the percentage of cell viability and increased the expression of TNF-α, a well-known pro-inflammatory mediator involved in ocular inflammation [38]. The results also revealed that the pretreatment with PAD428-FR2 0.01% determined a marked increase in cell viability and a decrease of TNF-α levels with respect to LPS-treated cells, demonstrating a good protective activity of fraction against the inflammatory stimulus. 

These data are in agreement with several in vitro and in vivo studies; for instance, Baci et al. (2019) reported that a polyphenol-rich extract from olive mill wastewater was able to induce a downregulation of pro-inflammatory pathways in prostate cancer cells [54]. Richard et al. (2011) evidenced that hydroxytyrosol was the major anti-inflammatory compound in aqueous olive extracts able to impair cytokine and chemokine production in murine macrophages stimulated with LPS [18]. In particular, Fuccelli et al. (2018) observed that hydroxytyrosol reduced the TNF-α secretion in LPS stimulated mouse model [17].

Based on the previous biological assays, 0.01% *w*/*w* of lyophilized PAD428-FR2 was used to formulate a novel nutraceutical ophthalmic preparation. Traditional ophthalmic liquid formulations are characterized by limited residence time in the eye due to lacrimal secretion and nasolacrimal drainage resulting in a low drug absorption (only 1–10%) and limited efficacy. The increase of viscosity of the formulation using biocompatible hydrophilic polymers with mucoadhesive properties represents one of the best strategies to prolong the residence time in the eye [55,56]. Indeed, several studies have reported that the water-base gels have several advantages over the traditional ophthalmic formulations, either in terms of enhanced therapeutic response or improved ocular bioavailability [57]. According to these considerations, in this study a combination of different hydrophilic polymers as Carbopol^®^980 and Pemulen™RT1-NF was employed to enhance the viscosity and obtain a hydrogel with a 3D polymeric network [58]. Hydrogel was prepared according to the protocol described in the “Materials and Methods” section, and the final composition is shown in Table 1. 

The ophthalmic formulation was clear without any suspended particles or impurities and showed a pH value equal to 6.7 which is considered physiologically compatible. As reported in the Materials and Methods section, the values regarding pH, appearance, osmolality, and hydroxytyrosol (%) were evaluated to assess the chemical and physical stability in the storage period. The results of stability study, shown in Table 4, revealed no significant changes with respect to hydroxytyrosol concentration (%), pH, osmolality, and appearance in samples stored at 25 ± 2 °C with 60% ± 5% R.H. for 3 months. 

Finally, we evaluated the ocular irritation of hydrogel using SkinEthicTM HCE model, composed of human corneal epithelial cells that forms a corneal epithelial tissue when cultured at the air–liquid interface in a chemically defined medium on a permeable synthetic membrane insert [39].

The percentage of cell viability evaluated by MTT assay was equal to 95.7 ± 5.3 for tissues treated with hydrogel. According to the viability classification prediction model, since the percentage of viability is more than 60%, the hydrogel can be classified as non-irritant.

### 3.5. Scaling up and Pilot Plant Development

A mandatory feature for a process aiming to be an applicative and possibly marketable development is economic sustainability. In order to evaluate this parameter and with the aim to test the scalability, reproducibility, and effectiveness of our protocols at a pre-industrial scale too, an automatized pilot plant for polyphenols extraction from OMWW was realized by using the optimized laboratory conditions. 

**Figure 12:**
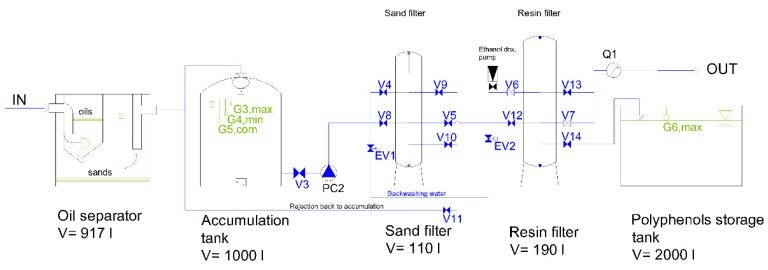
Scheme of the developed pilot plant prototype.

In Figure 12 is the schematized pilot plant consisting of the following steps: de-oiling and sand removal through a settler and an oil-separator, sand filtration, adsorption process through Purosorb™PAD428 and discharging of the not retained dephenolized fraction (FR1), and finally desorption of the polyphenolic mixture (FR2) stored in a 2000 L accumulation tank by eluting with water\ethanol 50:50. A mixture of ethanol/isopropanol, 85:15, instead of pure ethanol, was used in the desorption phase because it represented the purest composition on the market for semi-industrial use.

The empty bed contact time, with the operating flow, was about 48 min, a sufficient time to reach the maximum adsorption, according to laboratory results.

Preliminary runs in the pilot plant (see Figure S9 of prototype in Supplementary material) showed that the various fractions have features tending to satisfactorily match the laboratory results but need to be furtherly optimized. This is shown in Table 5, which compares olive mill wastewaters and fractions FR1/FR2 characteristics, obtained from the treatment in the pilot plant. It is to be noted that the difference in composition between lab and scale-up could partly be explained by the different purity of used eluents.

The FR1 showed a reduced content in polyphenols (more than 50%), and a COD abatement of about 32%. This sugar-enriched fraction was used to develop novel cosmeceutical formulations as described in a previous work [30]; alternatively, it is possible to imagine for this fraction a final oxidative stage (aerobic microbial digestion) able to furnish clear depolluted water and, as a secondary product, some sludge that could find easy application as compost in agriculture due to its rich organic content. A further reduction of polyphenols content would help the potential subsequent biological sludge treatment, with the addition of urea and ammonium phosphate, to achieve the correct nutritional balance. After evaporation of the solvent, the residues of the FR2 alcoholic extract were analyzed by HPLC (chromatogram not reported): The main components, analogously to the lab-scale process, were tyrosol and hydroxytyrosol, with an average polyphenolic recovery of 22%, and a hydroxytyrosol purity of 40%. The lower polyphenolic recovery value (if compared to laboratory results) needs to be improved and investigations regarding the optimization of the scaling up parameters, especially flow rate and contact time, are actually in progress, but in general these firsts pilot plant operation experiments demonstrated that expected results are not too far from being reached.

In the pilot plant prototype, the total installed power (calculated as the sum of the nominal powers of all power-consuming sections) is about 1 kW, and considering a ten cycle operation, a preliminary economic balance was assessed, as illustrated in Table 6. Operational only costs were included, while any investment cost was excluded. Noteworthy, by recycling ethanol, that represents one of the major costs in the procedure, a substantial reduction of the total cost could be achieved, but one of the main objectives of subsequent studies should be a better regeneration of the resin and/or the extension of its useful life. 

## 4. Conclusions

The described approach allows the ability to turn a highly polluting waste into valuable fractions, which can potentially be considered as raw starting materials for pharmaceutical/nutraceutical applications. A pilot plant prototype was realized and a preliminary economic balance calculated, showing the feasibility of the process at a pre-industrial level. In vitro biological assays were performed on the obtained polyphenolic fraction to study its cytotoxicity and anti-inflammatory/antioxidant activities, and the results permitted the ability to formulate a novel ophthalmic hydrogel with promising features that of course need yet to be more deeply characterized in view of a commercialization plan.

## Figures and Tables

**Table 1:** Composition of the ophthalmic hydrogel.

Ingredients	Composition (%)
PAD428-FR2	0.01
Carbopol^®^980	0.20
Pemulen™RT1-NF	0.01
EDTA	0.02
Glycerol	1.15
Na_2_HPO_4_·12 H_2_O	0.25
NaOH	0.07
Purified water	Up to 100

**Table 2:** Chemical characterization of olive mill wastewater (OMWW) and fractions.

Characterization	OMWW	PAD428-FR1	PAD900-FR1	PAD428-FR2	PAD900-FR2
Total Nitrogen (mg/L)	350.0 ± 3.4	318.0 ± 3.1	320.0 ± 3.2	1.0 ± 0.2	1.0 ± 0.2
Total Phosphorous (mg/L)	186.0 ± 2.1	144.0 ± 2.3	149.0 ± 2.1	0.5 ± 0.01	0.5 ± 0.01
COD (g/L)	73.65 ± 1.43	44.80 ± 1.34	44.02 ± 1.55	20.42 ± 1.25	21.07 ± 1.29
BOD5 (g/L)	38.44 ± 2.12	26.42 ± 2.05	26.40 ± 2.19	15.60 ± 1.99	15.33 ± 1.92
Total Polyphenols (g/L)	5.20 ± 0.14	0.40 ± 0.04	0.38 ± 0.04	4.12 ± 0.11	3.94 ± 0.12
Hydroxytyrosol (g/L)	1.10 ± 0.08	-	-	0.90 ± 0.06	0.85 ± 0.06
Tyrosol (g/L)	0.14 ± 0.02	-	-	0.10 ± 0.02	0.08 ± 0.01
Total Sugar (g/L)	34.00 ± 2.18	24.22 ± 1.14	23.75 ± 2.09	4.70 ± 0.59	4.82 ± 0.32

Mean values (*n* = 9) ± SD were calculated.

**Table 3:** Freundlich and Langmuir isotherms constants for total polyphenols adsorption on Purosorb™PAD428 resin.

Freundlich Isotherm			Langmuir Isotherm		
*R* ^2^	*N*	*K_F_*	*R* ^2^	*Q* ^0^	*K_L_*
		((mg/g)(L/mg) ^1/n^)			L/mg
0.962	2.0	1.0	0.993	47.7	0.02

**Table 4:** Stability study performed on ophthalmic hydrogel stored at 25 ± 2 °C with 60% ± 5% R.H. for 3 months.

Months				
	0	1	2	3
Hydroxytyrosol (%)	100	99	98	98
pH	6.7 ± 0.1	6.7 ± 0.1	6.6 ± 0.1	6.7 ± 0.1
Osmolality	150.0 ± 1.0	153.0 ± 1.0	155.0 ± 1.0	155.0 ± 1.0
Appearance	transparent	transparent	transparent	transparent

Mean values (*n* = 9) ± SD were calculated.

**Table 5:** Characterization of eluted fractions from preliminary pilot plant tests.

Characterization	OMWW	PAD428-FR1	PAD428-FR2
Total Nitrogen (mg/L)	350.0 ± 3.4	204 ± 1.6	1.0 ± 0.2
Total Phosphorous (mg/L)	186.0 ± 2.1	97 ± 1.4	0.5 ± 0.1
COD (g/L)	73.65 ± 1.43	50.60 ± 1.93	20.42 ± 1.64
BOD5 (g/L)	38.44 ± 2.12	19.82 ± 2.24	15.60 ± 1.45
Total Polyphenols (g/L)	5.20 ± 0.14	2.10 ± 0.09	1.12 ± 0.08
Hydroxytyrosol (g/L)	1.10 ± 0.08	not detected	0.45 ± 0.07
Tyrosol (g/L)	0.14 ± 0.02	not detected	0.06 ± 0.01
Total Sugar (g/L)	34.00 ± 2.18	23.50 ± 3.11	3.55 ± 3.38

Mean values (*n* = 9) ± SD were calculated.

**Table 6:** Economical balance of the pilot prototype for ten-cycle operations.

Cost Item	Quantity (Units)	Unit Cost (€)	Total Item Cost (€)
Adsorbent (kg)	32	38.00	1216.00
Ethanol (L)	500	1.80	900.00
Manpower (hours)	2	25.00	50.00
Wastewater treatment (m^3^)	2	0.80	1.60
Energy (kWh)	15	0.10	1.50
Mains water (m^3^)	2	0.30	0.60
		Total 10-cycles cost	2169.70
Average polyphenolic extract production for 10 cycles (kg)			2
Estimated cost per kg of extract (€/kg)			1084.85

## Supplementary Materials

The following are available online at https://www.mdpi.com/2076-3921/8/10/462/s1, Table S1: Metals analysis of Cerasuola-OMWW and fractions performed by ICP-MS, Table S2: Biophenols in Cerasuola-OMWW and PAD428-FR2 performed by HPLC-DAD analysis, Figure S1: Pictures of unabsorbed fraction eluted with water (PAD428-FR1, left) and fraction eluted with water/ethanol (50/50) solution (PAD428-FR2, right), Figure S2: Chromatogram of PAD428-FR1 at 280 nm, Figure S3: Chromatogram of PAD900-FR1 at 280 nm, Figure S4: Chromatogram of PAD550-FR1 at 280 nm, Figure S5: Chromatogram of PAD900-FR2 at 280 nm, Figure S6: Chromatogram of PAD550-FR2 at 280 nm, Figure S7: ^1^H-NMR spectrum of PAD428-FR1 exhibited only signals in the typical regions of the alkylic (1–2 ppm) and heteroalkylic groups (3–4 ppm), confirming the absence of polyphenols and giving indications about the presence of carbohydrates, Figure S8: ^1^H-NMR spectrum of PAD428-FR2 exhibited the characteristic signals of aromatic compounds (around 6.0–7.5 ppm) confirming the aromatic nature of the compounds in the mixture. Alkylic (1–2 ppm) and heteroalkylic groups (3–4 ppm) are still present and can be assigned to the sugar moiety of the glycosylated phenols, Figure S9: Picture of the pilot plant.
