# Supplementary material for: Polyphenolic Fraction from Olive Mill Wastewater: Scale-Up and in Vitro Studies for Ophthalmic Nutraceutical Applications
Source: Antioxidants (Basel). 2019 Oct 8;8(10):462. doi: 10.3390/antiox8100462 (PMC6827079; doi:10.3390/antiox8100462)
Supplement: Supplementary file 1 [file antioxidants-08-00462-s001.pdf]

Table S1. Metals analysis of Cerasuola-OMWW and fractions performed by ICP-MS

Metal (mg/kg)	Cerasuola-OMWW	PAD428-FR1	PAD428-FR2
**Sb, Hg, As, Ag, Cd, Se, Bi, Be**	< 0.01	< 0.01	< 0.01
**Mo**	0.01	0.01	< 0.01
**Pb**	0.03	0.01	< 0.01
**Al**	0.30	0.28	0.02
**Cr**	0.02	0.01	< 0.01
**Co**	0.02	0.02	< 0.01
**Cu**	0.60	0.39	0.12
**Zn**	3.00	2.69	0.07
**Fe**	1.99	1.52	0.11
**Mn**	2.17	1.92	0.04
**Ni**	0.11	0.09	0.01
**Na**	303.34	274.74	4.10
**K**	7379.34	6850.37	6.36
**Ca**	61.74	51.03	2.91
**Mg**	240.56	216.87	4.05

Method according to Di Mauro et al., (*Food Funct*., 2017,**8**, 4713-4722)

Table S2. Biophenols in Cerasuola-OMWW and PAD428-FR2 performed by HPLC-DAD analysis

**Biophenol (mg/l)**	**Cerasuola-OMWW**	**PAD428-FR2***	**PAD428-FR2°**
**Gallic acid**	-	-	-
**Pyrocatechol**	6.59±0.08	5.34±0.07	3.26±0.07
***p*-Hydroxybenzoic acid**	-	-	-
**4-Hydroxyphenylacetic acid**	traces	traces	traces
**Caffeic acid**	9.12±0.11	8.55±0.12	5.88±0.08
**Floretic acid**	21.17±0.21	18.23±0.19	10.61±0.09
**Verbascoside**	traces	traces	traces
***p*-Coumaric acid**	4.27±0.13	3.28±0.06	traces
***Trans*-ferulic**	-	-	-
**Oleuropein**	14.32±0.15	12.91±0.12	7.32±0.11

* PAD428-FR2 obtained in laboratory; ° PAD428-FR2 obtained by process scale-up.
Mean values (n=9) ± SD were calculated

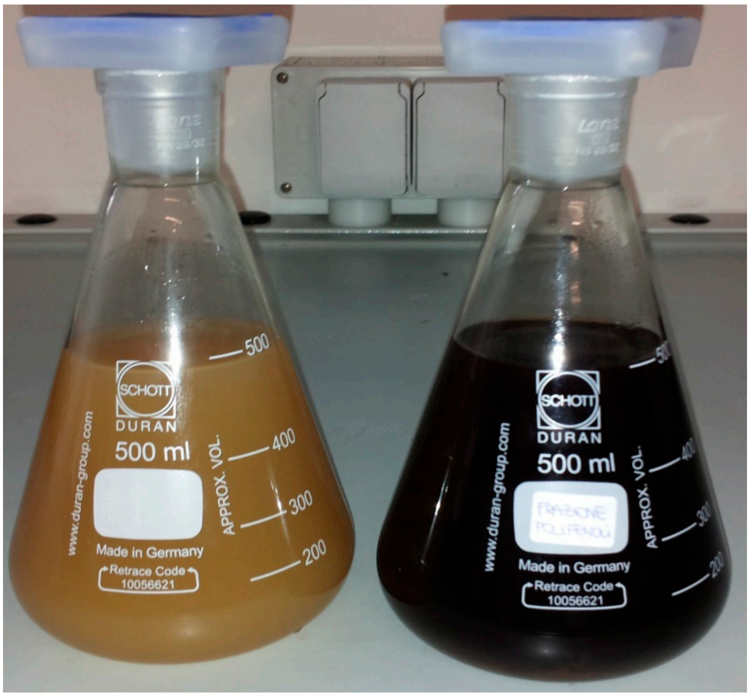

Figure S1. Pictures of unabsorbed fraction eluted with water (PAD428-FR1, left) and fraction eluted with water/ethanol (50/50) solution (PAD428-FR2, right).

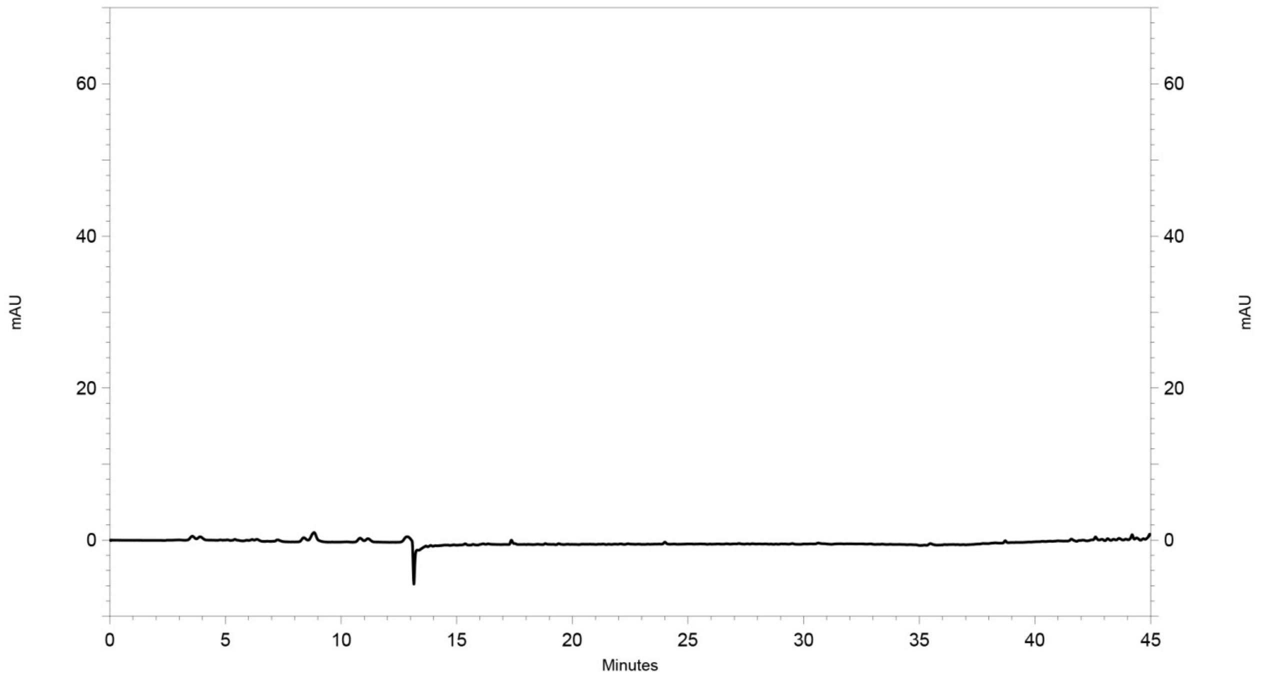

Figure S2. Chromatogram of PAD428-FR1 at 280 nm.

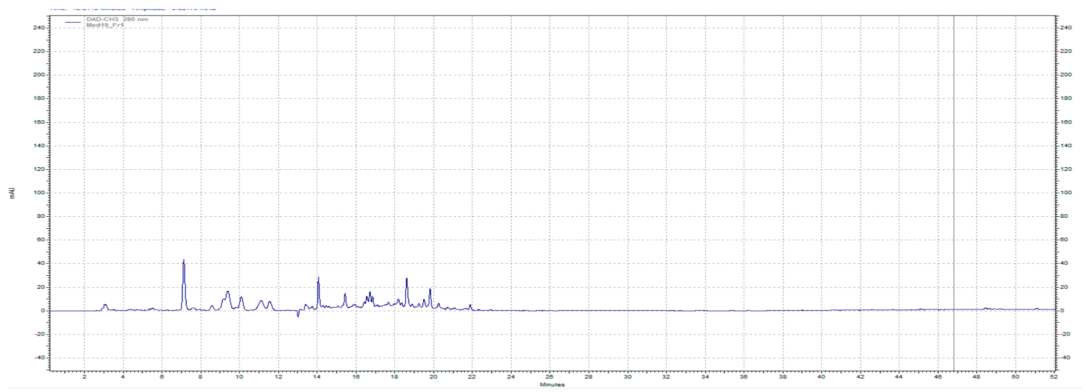

Figure S3. Chromatogram of PAD900-FR1 at 280 nm.

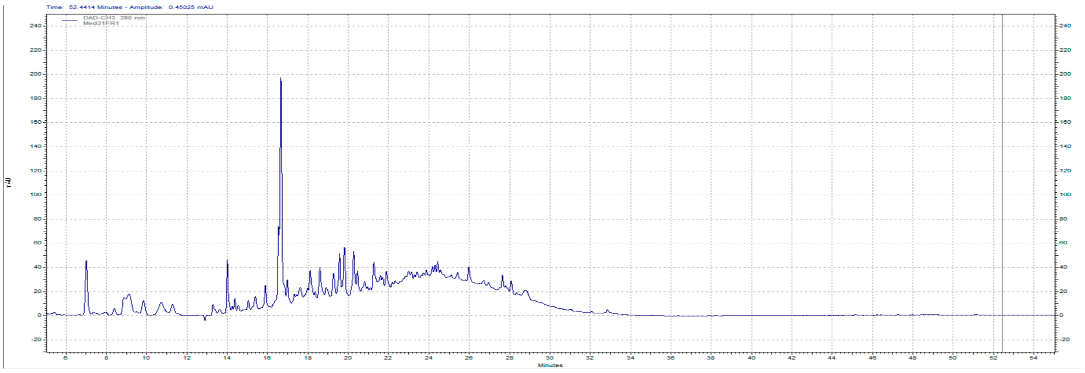

Figure S4. Chromatogram of PAD550-FR1 at 280 nm.

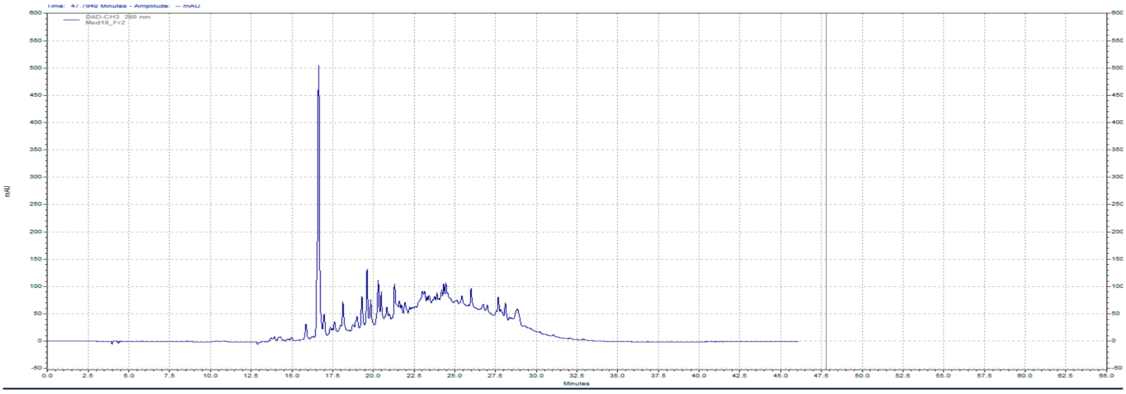

Figure S5. Chromatogram of PAD900-FR2 at 280 nm.

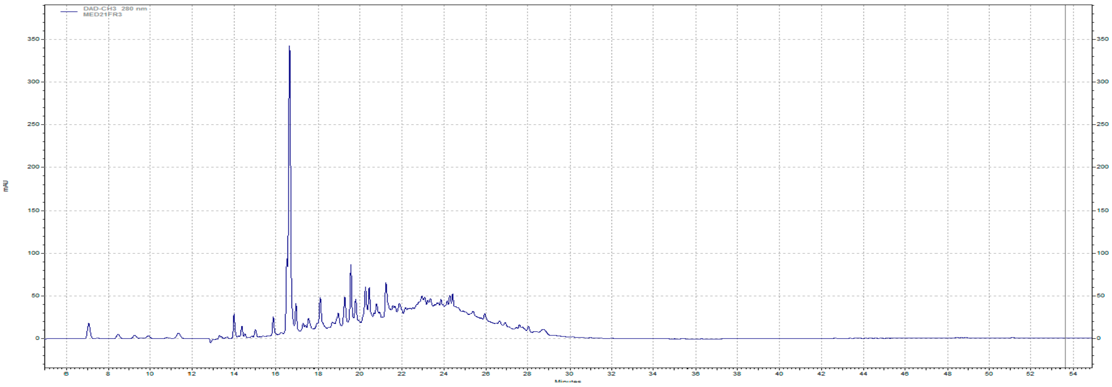

Figure S6. Chromatogram of PAD550-FR2 at 280 nm.

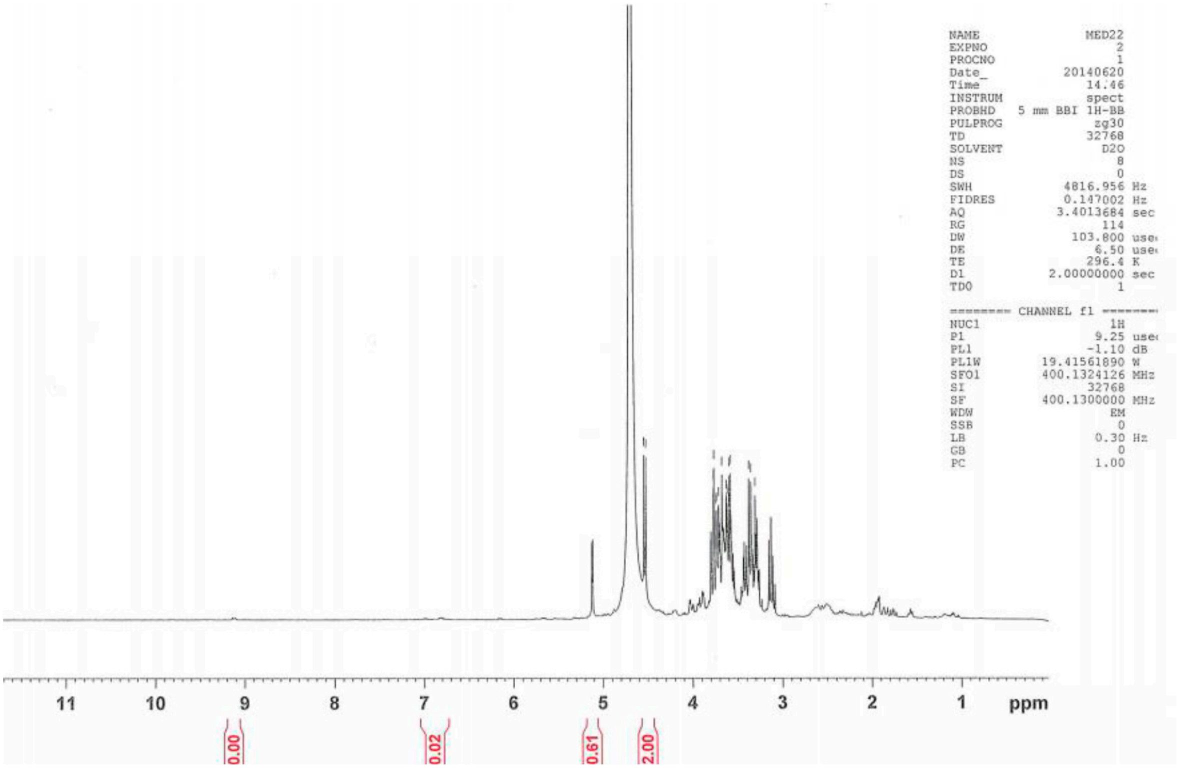

Figure S7. ^{1}H-NMR spectrum of PAD428-FR1 exhibited only signals in the typical regions of the alkylic (1-2 ppm) and heteroalkylic groups (3-4 ppm), confirming the absence of polyphenols and giving indications about the presence of carbohydrates.

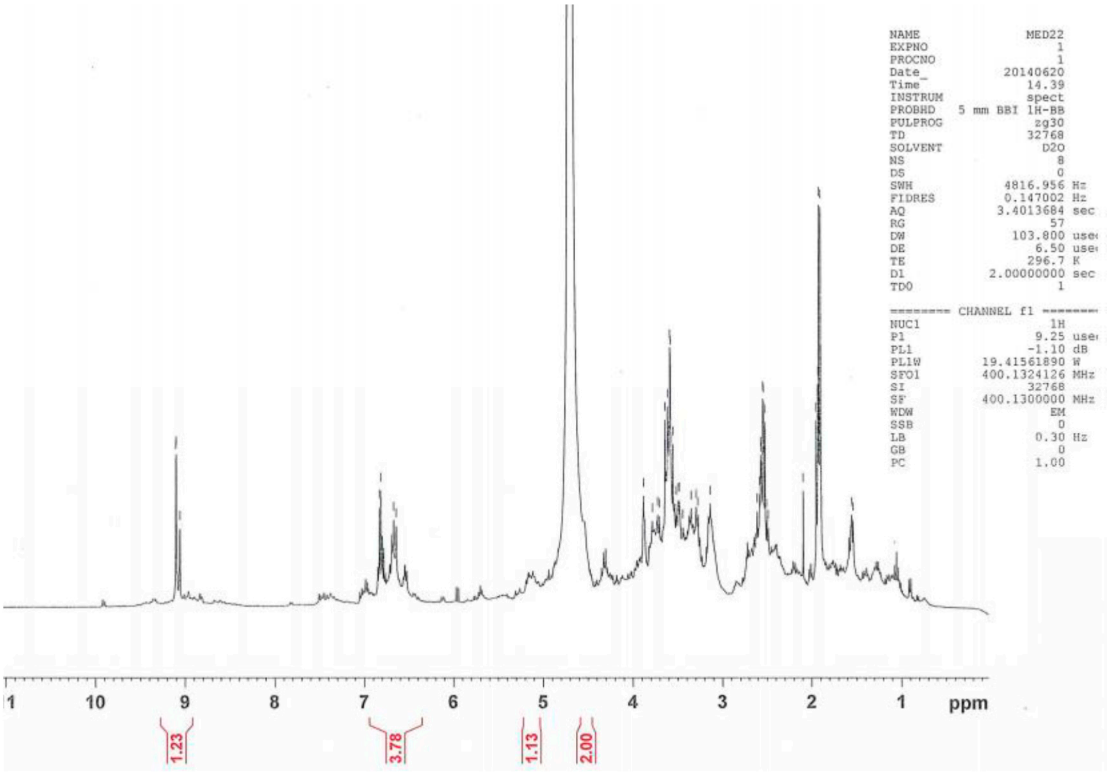

Figure S8. ^{1}H-NMR spectrum of PAD428-FR2 exhibited the characteristic signals of aromatic compounds (around 6.0-7.5 ppm) confirming the aromatic nature of the compounds in the mixture.

Alkylic (1-2 ppm) and heteroalkylic groups (3-4 ppm) are still present and can be assigned to the sugar moiety of the glycosylated phenols.

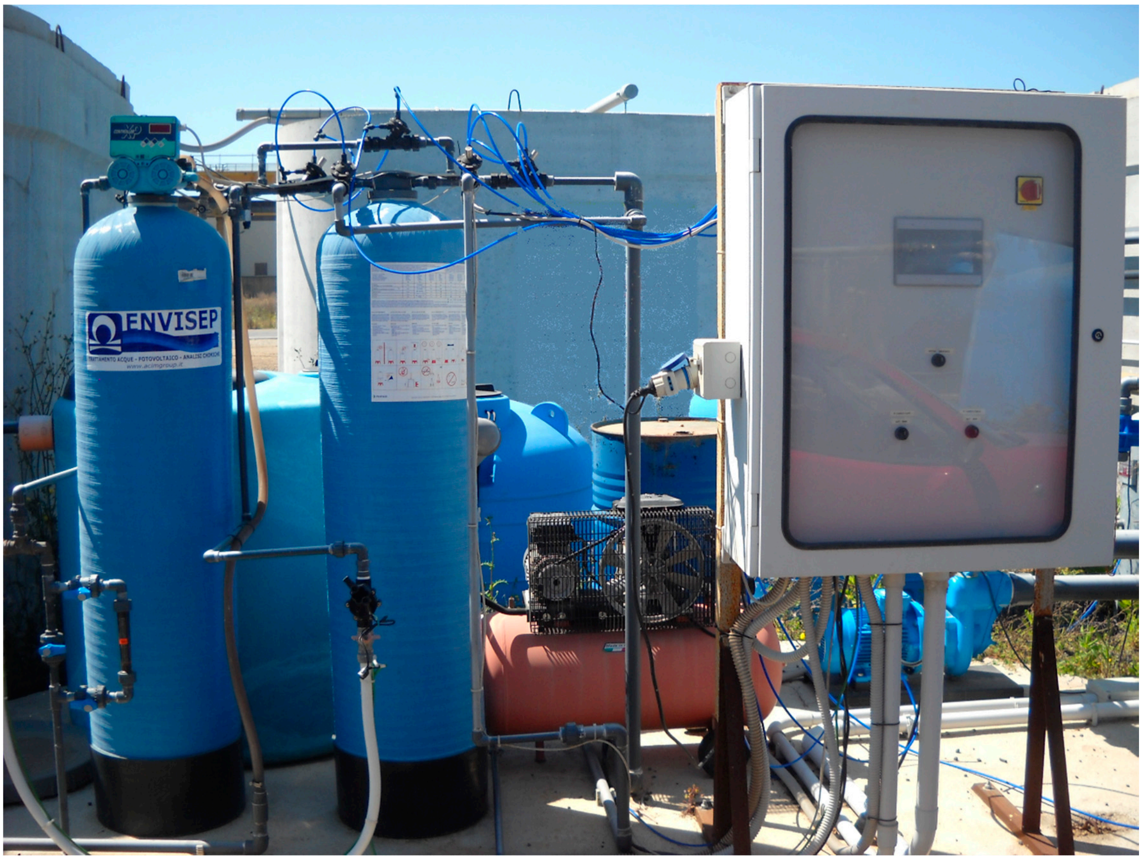

Figure S9. Picture of the pilot plant.
